# Atlanta Society of Medicine

**Published:** 1896-07

**Authors:** W. L. Champion

**Affiliations:** Secretary; Atlanta, Ga.


### SOCIETY REPORTS.

---

### ATLANTA SOCIETY OF MEDICINE.

ATLANTA, GA., March 3, 1896.

In the absence of both the President and Vice-President, Dr. V. O. Hardon was called to the chair.

The regular order was a discussion of "Normal Labor."

DR. F. B. MCRAE: *Previous preparation of the patient, etc.* As I consider it, the preparation should begin at least a month before confinement. I see that the secretory and excretory organs are doing their work, and that the food is of the right kind and is taken up by the system properly. This would bring us up to the confinement, and would again be sure that the bowels and abdominal organs were in proper condition. The main thing is to have the kidneys in order, and we would expect trouble from them if they are not looked after. I always examine the urine to ascertain if there is any albumen or anything of that kind. To correct that I use bromide of sodium, and also a little chloroform. Usually the bromide is sufficient. When the patient comes up to confinement, if possible, I have prepared a room distant from the street, with plenty of light and ventilation, and with as little in the room as possible. I prefer a cotton pad on the bed, and I do not believe in taking a pad from one bed to another. I use a different pad for each patient, and destroy it afterward. I have an oil-cloth over the pad. I see that everything is as simple as possible, and eliminate as far as possible all heavy diet for several days previously. In regard to nurses, I have a great deal of trouble with them. The worst trouble is that some families who have had, still have, and intend to have, some old colored nurse. These I find very hard to do anything with, as they have very set ideas. I always try to have a couple of white nurses that I can depend upon. I like to have them see that the clothing, bed linen, and everything else is ready, and to see to the means of getting rid of the excretions. I

do not give vaginal douches, but of course I see that the body is bathed properly. The secretions of the vagina are there to facilitate labor, and should not be removed.

DR. E. H. RICHARDSON: *Management of the first stage of labor.* The duty of the accoucheur in the management of the first stage of a normal labor is one of expectancy and watchfulness. Therefore the duty assigned to me to open this discussion on the management of the first stage of labor is an exceedingly simple one, and necessarily circumscribes our field of observation. It very often happens that we see our obstetrical cases for the first time during this stage of labor. It is perhaps a supererogation to say that the obstetrician of to-day should be equipped and prepared to meet the gravest problems incidental to the most untoward labor. His armamentarium should contain chloroform, ether, a thumb-lancet, veratrum viride, morphine, a hypodermic syringe, a silver and elastic catheter, Elliot's forceps, silk thread, a needle holder, tablets of mercuric chloride, iodoform gauze, carbolic acid, a few large sponges, Barnes's dilators, a Davidson's syringe, brandy, etc. Supplementary to this list, perhaps a modern surgeon's pocket-case of instruments will be sufficient to meet the ordinary requirements of most contingencies that might arise. Regardless of the view any one has taken in the past concerning the value of antiseptic midwifery, its importance can no longer be questioned. The diminished mortality since its principles have been adopted in the maternity hospitals of Europe and America is conclusive testimony in its favor. The clothing and person of the accoucheur should be eminently clean. His hands and arms having been well washed with soap and nail-brush, they should be immersed in a one to two thousand solution of bichloride of mercury. If the information has not been previously obtained, the first duty of the obstetrician is to obtain a correct history of the case. He should quickly satisfy himself as to the character of the subject he has before him, whether or not the patient is in a fair state of health, or the contrary; the family history, history of previous labors, and her present condition should be ascertained. The hands emerging from the bichloride solution of mercury may now be lubricated with a three per cent. solution of carbolic acid and glycerine, and a vaginal examination, coupled with external palpation, is now instituted. By the combined method the exploration should be sufficiently thorough to ascertain the presentation of the child, its condition, whether living or dead, the condition of the pelvis, uterus, amount of dilatation, condition of bladder, perineum, and rectum. In European hospitals, on admission into the maternity department, each patient's pelvis is examamined and measured, but in England and America pelvic deformities are so rare that this precaution is not deemed necessary. The woman should be directed to empty her bladder at regular intervals, and the nurse should flush the rectum with a copious enema and see that it is well emptied. It has been determined that the patient is at the end of the utero-gestation, and that the pains are genuine labor pains. False pains are distinguished from true pains of labor by their location in the abdomen, irregularity in time and severity, the absence of "show," and the absence of dilatation of the cervix uteri. The pains of the first stage of labor are lancinating, cutting, and teasing. Each pain is the expression of a uterine contraction which begins at the internal os, and is nature's method of dilatation, and gradually extends outwards until the *vis a tergo* forces open the external os, the diameter of which is increased and its lips thinned out until large enough for the reception of the body of the child. It has been well said that "the essential prerequisites of the skillful and successful obstetrician are competency, deliberation, alertness, promptness, and self-possession. Should our investigations reveal to us that we are confronted with such grave complications as placenta previa, an abnormal presentation, eclampsia, stenosis of vagina or uterus, or a dead child, interference should be immediate. A supine, waiting attitude, in view of the evil consequences to mother and child now, would be both ignorant and culpable. But if everything relating to mother and child in this stage is normal the accoucheur has but little to do. When dilatation is complete, the dilatation being from three to three and a half inches, the amniotic sac usually bursts spontaneously, and the second stage of labor is ushered in by a gush of water which is collected by the large sponges placed at the osteum vagina for the purpose. Should the bag of waters not break spontaneously after they have subserved their purpose, if the finger nail, during the acme of a pain, is pressed firmly against the distended membranes, the sac is easily ruptured. Up to this time the patient has not been restrained from walking, sitting, or regulating her own movements. She has been allowed to eat and drink *ad libitum*, and the physician who has not heretofore been constantly present with the patient now has her placed in bed, and as the pains have become expulsive and more frequent, she is directed to aid the expulsive movements by bearing down, and during the pains chloroform is administered. In this way chloroform may be given with impunity. Bedford Brown is authority for the statement that in all medical literature not more than forty cases are reported of deaths from anesthetics administered during labor.

DR. J. W. DUNCAN: *Management of the second stage of labor.* I had no idea at all of leading the discussion on any point. Dr. Richardson has said more than I thought could be said on the first stage of labor; but he left out one thing I would use, and that is the chloroform. I get better results in the first stage than in the second. It facilitates the dilatation of the os; and after the first stage has passed, if the woman is not very nervous and does not make unusual complaint, I do not give the chloroform. In the second stage and after the membrane has ruptured, we ascertain the position of the head, and if there is any deviation from the normal we try to rectify it. After it has engaged the pelvis there is very little to do until the head begins to press upon the perineum. Then I give some aid in dilating the perineum. It may not benefit in some cases. As the head presses down firmly and dilates the perineum, I retard the advance of the head to some extent. In some cases this is absolutely necessary. Where the pains are very strong I think it best to retard the progress of the child and let it be distended gradually. At the first presentation I elevate by pressing firmly just below the coccyx. In some cases I have found it necessary to pass two fingers into the rectum and in that way bring the head forward and have hurried labor. There are some cases in which the cord is wound around the neck one, two, and sometimes three times. Then when the pain subsides the head recedes back to where it commenced. I have had cases where the child would advance and recede for an hour or more, and I would then pass two fingers into the rectum and get them under the chin and hold them that way, and at the next pain the head would be born and I would then release the cord from around the neck. This is not normal, but is so frequently the case that it is not unusual. My plan has been not to be too hurried about the use of forceps. After the first stage has passed and the second stage has commenced in the usual way with the exception of sufficiently strong pains, I have been inclined to wait three or four hour before using the forceps, and I believe that is the best plan in most cases. In my mind, about four hours should elapse before using the forceps. In some cases I would not wait so long, but would be governed according to the case. The delivery of the shoulders is usually not a very difficult thing. In some cases if the shoulders are delayed it is well to pass the finger into the axilla and bring down one arm and then the other. Where the shoulders are large and the pelvis contracted we have some difficulty, but I have not had many cases where it was necessary to deliver an arm before the shoulders were passed.

DR. VAN GOIDTSNOVEN: *Management of the third stage of labor.* The third stage of labor is occupied in the expulsion of the placenta. In a spontaneous, normal, or natural labor it would seem that the task or duty of the obstetrician is plain, and that is, to let nature take its own course, labor being a physiological act. But it is evident that he should be on his guard to the last; for although the two first stages may have passed normally and satisfactorily, he should remember that "all is well that ends well," and that the third stage is liable to multitudinous accidents and complications. He cannot, therefore, at the incipiency of the last stage congratulate himself upon a complete success predicated upon the favorable conclusion of the first two stages. Immediately after the delivery of the child, the woman is either plunged, as it were, into a momentary interval of relief and repose, or somewhat disturbed by the fear of new pains. I have often thought that this suspension is due to a momentary paretic condition of the uterine walls consequent upon the violent contracting pains that have been wrought in the fetal expulsion. The length of the delay is generally in inverse ratio of the physical strength and nervous condition of the patient. Experience shows that if the expulsion of the after-birth is left to the uterine and vaginal contractions, in very few cases this spontaneous delivery is effected with rapidity; whilst in others delay is more or less required, one, two, and even five or six hours after the birth of the child. In rarer cases spontaneous expulsion cannot and will not occur, and artificial delivery having become necessary shall be rendered the more difficult proportionately to the time spent in waiting, retraction of the internal orifice having then taken place with considerable energy. It follows that delivery must always be aided though not forced. Certain delay is necessary for coagula to form within the uterine sinuses. This requires a reasonable time, from fifteen to twenty minutes. Unfortunately it is too often the case that the practitioner's attention at this stage of labor is fixed mainly on the hasty delivery of the placenta, even before the contractions of the womb take place and close the uterine sinuses and mouths of vessels, thus exposing the patient to serious and even fatal loss of blood. Extraction of the after-birth is contrary to the laws of natural and spontaneous labor, and it is too often resorted to by the practitioner who is in a hurry to get through. In pulling and jerking at the placenta he uses the *vis a fronte.* Nature does not pull but contracts and uses the *vis a tergo.* In view of the above mentioned danger, the practitioner's method is hardly anything short of criminal temerity. For it may produce, first, copious hemorrhage from partial detachment of the placenta; second, inversion of the uterus; third, separation of the cord from the placenta; fourth, irregular or hour-glass contraction of the uterus; fifth, septic poisoning and phlebitis caused by the retention of placental fragments, portions of membranes and clots which may remain behind. Ordinarily, as soon as the child is expelled, I pass my hand over the abdomen of the patient to ascertain first, if there be another child; second, if there be fibrillary contractions of the uterus. In the latter case the womb forms a firm rounded mass lying in the lower part of the abdominal cavity. I then feel satisfied that placental delivery is soon to take place and if the case be left to nature a miniature labor readily ensues. Through retraction of the internal surface of the placenta its attachments are easily separated; this mass remains in the cavity of the uterus as a foreign body. Pains immediately follow, and the placenta is spontaneously expelled from the uterus into the vagina or even externally. It is claimed by some authorities that when the placenta is drawn out in the way too generally practised, it obstructs the aperture of the os and acting like a piston of a pump, produces a vacuum and tends to promote hemorrhage. In the majority of cases, when the after-birth is detached from the uterine walls it either lies on the os or in the upper portion of the vagina, and is likely to remain there even for several hours, but for the assistance of the obstetrician. When retained in the uterus after its detachment, the placenta is buttoned, as it were, within the orifice. It should be dextrously unbuttoned by bringing its edge to the button-hole as you would do with your coat button. If the after-birth be retained in the vagina, a slight twisting of the cord around the fingers of the right hand whilst the fore and middle finger of the left hand are passed up to the insertion of the cord and used as a pulley, is all that is sufficient to make a slight traction and bring the after-birth away. Before extracting the placenta entirely, it is well to turn it around several times in order to twist the membranes which in this case are so liable to be torn, and consequently to be left behind in greater or less portions, which might occasion serious and complicated disorders.

Immediately after the delivery of the placenta and secundines, I douche the vagina and frequently the uterus with half a gallon of warm water to which I have previously added an ounce of Darby's prophylactic fluid. Any portion of placenta or of membranes or clots which may remain behind is thus disinfected and induced to leave. Should the placenta not come away within a reasonable time after the birth of the child, I use Cazeaux's method, which he calls "expression." To effect "expression," says Cazeaux, the physician should sit by the bedside with the hand on the uterus in order to secure contraction and prevent distention. The fundus should be grasped in the hollow of the left hand, the ulnar edge of the hand being well pressed down behind the fundus, and when the uterus is felt to harden strong and firm pressure should be made downwards and backwards in the axis of the pelvic brim. If this maneuver be properly carried out and sufficiently firm pressure made, in almost every case the uterus may be forced to expel the placenta into the bed along with the coagula that may be in its cavity. Another *modus operandi* which has proven highly satisfactory and successful is the application of Faradic electricity, the positive electrode being applied immediately below the umbilicus and the negative to the os. Strong contractions immediately follow, and delivery almost invariably results. When the placenta or any portion thereof cannot be moved in the ways just mentioned, the case ceases to be one of normal labor."

Dr. J. E. Sommerfield: *After-treatment of labor.* With the expulsion of the placenta the after-treatment begins. The hemorrhages should be prevented, usually by rubbing the uterus from the abdominal walls. When all danger of hemorrhage ceases the places are washed and the patient placed in a clean bed. The external parts are examined, and if lacerated I sew them up. Then the pad is placed before the vulva to receive the discharge. After the cord has been tied and severed, the child is usually greased with lard or oil and washed in a bath of ninety-eight degrees temperature, and examined for any malformation that may be present. Then the room is darkened and the patient allowed to sleep. I usually allow them to sleep from six to ten hours. When they awake I urge the patient to pass urine. Should the quantity be very small, or the patient unable to pass any, a small catheter is employed, first washing the external parts. If the patient is unable to void urine, this is done two or three times a day. The external organs should be washed every day. The vaginal injections are sometimes given, but in normal cases I never employ these. After ten or twelve hours, after the patient has had a refreshing sleep, I usually have the child to nurse. No matter how small amount it receives, it is better than artificial food. I then let it sleep. I have the child to nurse every few hours but never let it nurse at night, so as to let the mother have as much rest as possible. I give liquid diet, and on the third day I give the laxative. The dressing of the patient will be painful until about the fourth day. Before nursing the child the nipples are thoroughly cleansed, and should they be painful sub-acetate of lead is used. As for visiting the patient, I make it a rule to go twice a day for the first few days, and after that once a day. The time the patient should remain in bed is according to the local discharge. It is usually from one to ten days; letting her sit up when not fatigued.

DR. DUNBAR ROY was requested to discuss *Ophthalmia Neonatorum.* He spoke as follows: One method of treatment consists in simply douching the vagina thoroughly with a one per cent. solution of creolin for two weeks before the birth of the child. This is easy of manipulation and the results are very satisfactory. They are just as good as those where Crede's method is used. His method is at the birth of the child to drop in a drop of nitrate of silver solution, ten grains to the ounce. This is no better than some other methods that have been tried. In fact, I have found that the dropping of a ten-grain solution is too strong to use in a child's eye, and also sets up a mild conjunctivitis and leaves the eyes subject to infection. One grain would be sufficient, and I found it just as good as you would want to use, although five grains does not set up an irritation. But I also used what I found to be just as good and somewhat better, and that was simply the washing off of the eye thoroughly with a spray of 1 to 5000 solution of bichloride of mercury. I have noticed cases which were not caused by opening the eyes in the uterus or the vagina, but by the material sticking to the lids, and which got into the eyes as soon as they were opened. Hence I tried the method of simply washing the eyes with the 1 to 5000 bichloride of mercury, and out of fifty cases we had only one case of ophthalmia neonatorum It was just as good a record as has been made by the use of nitrate of silver. My experience has been that the vaginal canal should be douched two weeks before the delivery with a solution of creolin, and at the birth to wash the eyes with the bichloride solution, and let some get into the eyes, but very little. When ophthalmia neonatorum once sets in the eye cannot always be saved.

DR. VIRGIL O. HARDON: The ground has been covered so completely that there is very little opportunity for me to say anything more. As far as concerns the management of normal labor during the labor, there is very little to be done. There are more sins of commission than omission, as has been said, but we never know when a case is going to be normal. More attention should be attached to the case before the labor comes on than at the labor. I think that the way to prepare a woman is to begin at puberty. I will not discuss this point however to-night, although it may be shown to have a very direct bearing on the subject. I always insist on the patient letting me know before the child is expected, as I want to begin with my patient as early as the fifth or sixth month. Those things which conduce to the general well-being of the mother will conduce to the success of labor. This includes proper diet, exercise, proper clothing, and strict attention to the condition of the bowels and the kidneys. Constipation is the bane of pregnant women. There is no invariable plan of treatment, for one woman would thrive on one treatment and another on a different treatment. We have to be governed by the idiosyncrasies of the patient. It is of prime importance that we know whether there is albumen in the urine. I think that puerperal eclampsia could be almost stamped out if every patient three or four months before the labor would get perfect action of the kidneys.

In a normal labor after determining the presentation I do nothing more for the patient until the rupture of the waters. I go to sleep if at night, and have the nurse to call me as soon as the rupture takes place. Then I make another examination and ascertain how near the head has approached the perineum, and if the pains are normal pains, I let them go on for a while and make an examination in half or three quarters of an hour. I think that considerable can be done in normal labors toward preventing the rupture of the perineum. I think that the suggestion of Dr. Duncan of preventing the head from coming too fast is a good one. Another important thing is to keep the head in a state of flexion until the occiput is out beyond the pubic arch. In the third stage of labor I believe in the same plan that we use all through labor. Let nature do the work. If we aid in the expulsion of the placenta, we should do it by pressure, as I do not think any harm can be done in that way unless we use severe force. There is nothing to be gained in getting the placenta out in two minutes, instead of one hour, except in saving time for the patient. If we shorten the third stage half an hour we have brought the patient into a comfortable position so much the sooner. After the placenta has been expelled I keep my hand on the womb to keep up the uterine contraction. I usually continue the pressure for an hour, which also prevents the accumulation of blood-clots. By doing this we diminish to a large extent the severity of the after-pains. About the after treatment there is not much to be said. I do not use any vaginal douches either before or after labor if the case be a normal one. The examining finger should always be surgically clean. There is one point of considerable importance. Whenever the patient empties the bladder or bowels I have her raised to an upright position in the bed. This gives the best possible drainage for the patient. When the patient is lying down the drainage is imperfect and may influence the possibilities of the retention of septic products in the vagina. I do not believe in auto-infection in labor. I think the germs are from without and their inward course is facilitated by the imperfect drainage. I put the child to the breast the first day, as I think it promotes uterine contraction. I believe also from observation that unless we put the child to the breast the first day there is a liability of its losing the instinct of nursing to a certain extent. I may be mistaken in regard to this, but I have seen some cases which seemed to confirm this view. In delivering the shoulders I deliver the anterior one first, and I have found that a valuable way to do this is to grasp the body of the child by the neck or the head, and to press the whole body backward against the perineum, pressing the posterior shoulder into the hollow of the sacrum until the anterior one is born. I think that laceration of the perineum occurs as often from the shoulders as from the head. It has been proven by watching closely and seeing that the head did not produce any laceration but after the child was delivered the perineum was found to be lacerated. I do not give chloroform in the first stage of labor. I have found that women bear the pains of the first stage better than those of the second stage. Although they complain more in the first stage, yet they talk more and appear to have more vitality, but not so in the second stage, which seems to exhaust them rapidly. I give chloroform in the second stage of labor, giving it only during the pains, but its effects sometimes persist between the pains. I aim to keep my patient absolutely unconscious when the head is passing through the vulva. I do not use a binder, but I do not discourage the use of it. My experience has been that it does not do any good or any harm, and most patients do not want it. If I had a patient with a womb which was inclined to relax I might use it, but even then I would have my doubts. I do not think we would get much pressure from the binder unless we had a thick pad beneath it. I think it is apt to displace the uterus downwards. I do not encourage the use of it. I have everything that is soiled changed and have everything clean about the patient, and have a clean napkin placed over the vulva to be changed as soon as soiled.

W. L. CHAMPION, M.D., *Secretary.*

